# Analysis of the therapeutic effect of the long-acting injectable form of aripiprazole in incarcerated adult males in Greece

**DOI:** 10.1192/j.eurpsy.2025.806

**Published:** 2025-08-26

**Authors:** E. L. Pasparakis, I. Mouzas, G. I. Detorakis, E. I. Koiliari

**Affiliations:** 1 Department of Psychiatry, General Hospital of Agios Nikolaos of Lasithi, Agios Nikolaos of Lasithi; 2 Department of Pathology, Laboratory of Alcohology of the Medical School of the University of Crete; 3 Department of Pathology, University General Hospital of Herakleion (PAGNI), Herakleion, Greece

## Abstract

**Introduction:**

The World Health Organization (WHO) estimates that in Western societies the incidence of psychiatric disorders is up to seven times higher in the prison population. Drug dependence is also a major destabilizing factor in this population. Our study is the first one in Greece with a focus group of incarcerated males which have comorbidity of emotional psychosis and Substance Use Disorder.

**Objectives:**

Hypothesis testing: “Aripiprazole LAI antipsychotic treatment is associated with improved quality of life and functionality in incarcerated patients with comorbidity of Bipolar disease - I (BD-I) and Substance Use Disorder (SUD)”.

**Methods:**

30 patients with BD I were prisoners at the Penitentiary of Neapolis of Lasithi of Crete (Greece). Median age was 36 years (all men). 76.67 % had comorbidity of bipolar disorder type I (BD-I) and alcohol use disorders. 80% had comorbidity of BD-I and cocaine use disorders. 93.3% had comorbidity of BD – I and Cannabis Use Disorder. All were medicated in prison by aripiprazole LAI 400mg/month. For the evaluation of our hypotheses the instruments WHOQOL-BREF questionnaire and the CGI-S scale were used. The quality of life and the functionality were compared in each patient, before the initiation of the LAI medication and during the active treatment period. The minimum of follow-up period was 6 months. Five cases of patients, who remained compliant with LAIs treatment after release from prison for a period of at least 6 months maintained a very good quality of life without ever getting into trouble with the law again.

**Results:**

In all 30 patients (imprisoned) of our sample, the CGI-S score with depot aripiprazole therapy administrated at least for 6 months decreased statistically significantly from 5.72 ±0.88 to 2.94±1.33 (Paired Samples Wilcoxon Signed Rank Test p-value<0.001). Additional, the quality-of-life scale score of these patients increased statistically significantly from 0.7 ±0.53 to 3.6±0.67 (Paired Samples Wilcoxon Signed Rank Test p-value<0.001) with depot aripiprazole therapy administrated at least for 6 months too.

**Conclusions:**

Aripiprazole LAI significantly improves the quality of life and functionality of patients with dual diagnosis of emotional psychosis and SUD in prison. Our study highlights that ensuring medication compliance, during the incarceration and after the release from prison, delays the time to reincarceration for this specific population or diminishes importantly the probability of the reincarceration.

**Disclosure of Interest:**

None Declared

